# Cerebrovascular Involvement in Systemic Sclerosis

**DOI:** 10.1002/acr2.70032

**Published:** 2025-04-14

**Authors:** Maurizio Cutolo, Tamara Vojinovic, Sabrina Paolino, Rosanna Campitiello, Vanessa Smith

**Affiliations:** ^1^ Laboratory of Experimental Rheumatology and Academic Division of Clinical Rheumatology, Department of Internal Medicine University of Genova Genoa Italy; ^2^ IRCCS Ospedale Policlinico San Martino Genoa Italy; ^3^ Department of Experimental Medicine (DIMES) University of Genova Genoa Italy; ^4^ Department of Internal Medicine Ghent University Ghent Belgium; ^5^ Department of Rheumatology Ghent University Hospital Ghent Belgium; ^6^ Unit for Molecular Immunology and Inflammation VIB Inflammation Research Center Ghent Belgium

## Abstract

Systemic sclerosis (SSc) is a chronic autoimmune rheumatic disease characterized by vascular damage, immune system dysregulation and fibrosis. The hallmark features include microvascular alterations and progressive tissue fibrosis, affecting skin, internal organs as well central and peripheral nervous system, adding to the disease's complexity and influencing overall outcomes. Of note, SSc has also been linked to macrovascular and cardiovascular involvement, including cerebrovascular damage as observed in stroke. Indeed, advanced neuroimaging is highly recommended for assessing cerebrovascular status in overt SSc to evaluate the complex interactions between cerebrovascular dysfunction and brain tissue damage and/or inflammation. Cerebral vasospasm detected by angiography, as well as an increase in subclinical cerebrovascular atherosclerosis observed by ultrasonography (carotid intimal medial thickness), are predictive for elevated stroke risk. Furthermore, a significant brain hypoperfusion detected by magnetic resonance imaging, along with white matter focal and/or diffuse signal abnormalities in SSc, have been found associated with concomitant peripheral microvascular damage detectable by “Active” and “Late” nail fold video capillaroscopy scleroderma patterns. Finally, the presence of calcifications in small arteries and arterioles found postmortem in the brain of SSc patients reinforces the hypothesis that SSc is associated with brain vascular remodeling. Furthermore, the current state of art shows an increased risk of cerebrovascular events in the SSc, confirmed by neuroimaging. Given the lack of updated comprehensive reviews on cerebrovascular involvement in SSc, we gathered the most relevant evidence on central nervous system damage, highlighting the underlying mechanisms, clinical implications, and potential advantages that neuroimaging may provide for its early detection.

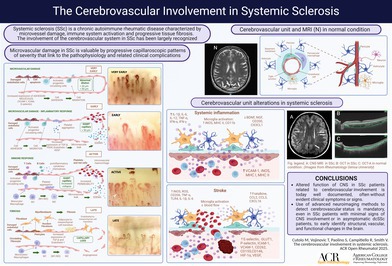

## Introduction

Systemic sclerosis (SSc) is a chronic autoimmune rheumatic disease (ARD) characterized by vascular dysfunction, immune system dysregulation, and progressive fibrosis.^1^ Indeed, widespread microvessel functional and structural abnormalities followed by progressive tissue fibrosis are the well‐characterized clinical findings in the majority of tissues and organs in patients affected by SSc.^2^

The central nervous system (CNS) involvement in systemic connective tissue diseases like rheumatoid arthritis (RA) or systemic lupus erythematosus (SLE) has been widely described during their development, based on both inflammatory and noninflammatory phenomena.^3^ CNS involvement in ARDs can vary depending on the underlying mechanisms. For example, vascular occlusion of the arterial bed seems to occur due to the effects of inflammatory reaction at the vessel wall (primary vasculitides) or due to immune complex deposition, which induces the expression of adhesion molecules and the migration of cells into the CNS through the blood‐brain barrier (BBB) disruption like in SLE.^3^

Indeed, a recent review highlighted the importance of performing advanced imaging techniques, like magnetic resonance angiography, in ARDs (SLE, Sjogren, RA, SSc, etc) to detect cerebrovascular involvement that can be underdiagnosed with traditional neuroimaging.^4^ Neuroinflammation caused by these events can lead to a disruption in neural networks and frequent CNS manifestations, especially in patients with SLE with concomitant presence of antiphospholipid antibodies. In the case of RA, cerebrovascular inflammation appears as a cluster of leptomeningeal infiltration of mononuclear cells around small vessels and necrotizing granulomas. This condition is relatively uncommon, although when present, it brings an increased risk of developing stroke or dementia in comparison with the general population.^4^ On the other hand, in SSc, microvascular damage of brain vessels is due to a complex endothelial cell (EC) dysfunction, leading to a typical noninflammatory microangiopathy, characterized by obliterative microvascular lesions and microvascular proliferation that alter the nervous system function during the disease progression.^5^

Although the hallmark features of SSc and specific therapies are traditionally and mainly concentrated on the skin, internal organs, and joints, several observations more recently indicated also the involvement of both the peripheral nervous system (PNS) and CNS, the latter significantly less frequent as the disease progresses.^5^, ^6^, ^7^ Further‐described clinical manifestations of CNS impairment in SSc include encephalopathy, seizures, vasculitis, depression, anxiety, headaches, and cognitive impairment.^8^ Notably, up to 61% of patients with SSc have been found to have mild cognitive impairment.^5^ Recently, ischemic stroke has been found to be the most frequent cerebrovascular accident among the more severe CNS pathologic implications in SSc.^5^ However, the pathophysiologic mechanisms and distinctive imaging aspects of CNS impairment in SSc are becoming better understood and certainly involve altered brain blood perfusion, as described from almost 30 years of investigations with different imaging techniques.^9^, ^10^ Because updated reviews regarding the cerebrovascular involvement and status in SSc are lacking, we collected and synthesized the current knowledge related to the CNS damage in SSc, reporting the suggested underlying mechanisms and clinical implications and presenting the new horizons and implications that CNS neuroimaging should further offer.

### The vascular involvement in SSc

Characteristic pathophysiologic findings that distinguish SSc from other ARDs are of pivotal importance and include the specific microvascular damage with associated tissue and organ progressive fibrotic processes.^11^, ^12^, ^13^ The damage and the apoptosis of ECs, with subsequent local production of chemokines by the immune cells activation, generate an inflammatory perivascular infiltrate and then possible tissue hypoxia, resulting in multiple organ involvement and related clinical manifestations in SSc.^14^

**Figure 1:**
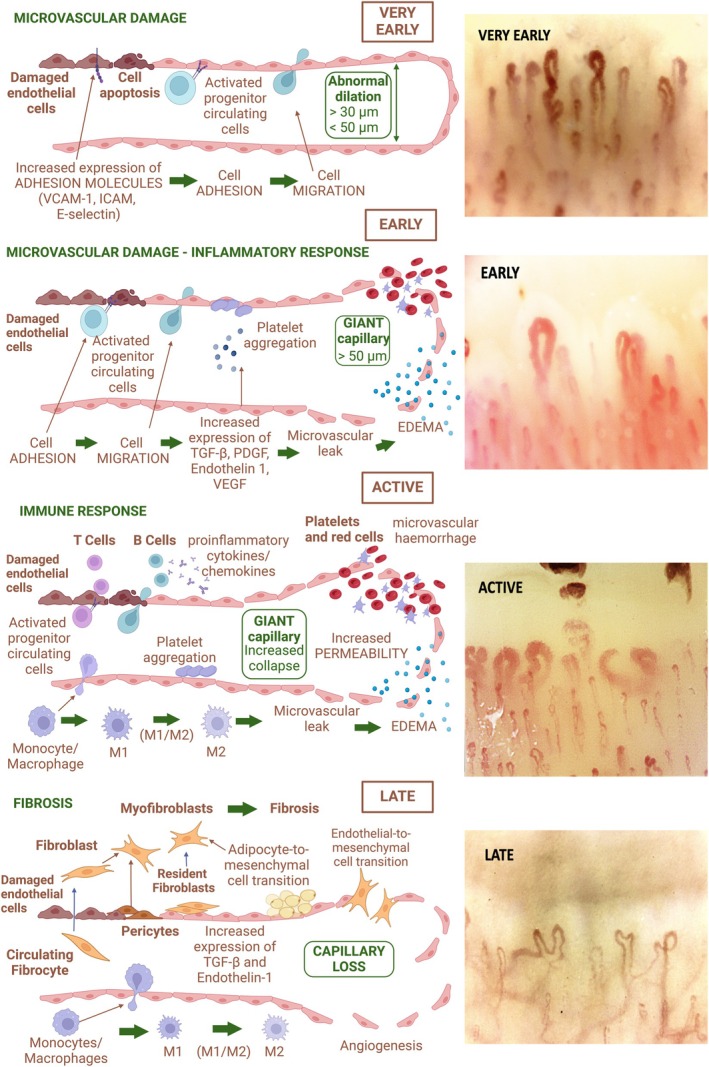
Nailfold videocapillaroscopy scleroderma patterns (“very early,” “early,” “active,” and “late”). Original figure created with BioRender.com. E‐selectin, endothelial‐selectin; ET‐1, endothelin 1; ICAM, intercellular adhesion molecule; M1, macrophage type 1; M2, macrophage type 2; PDGF, platelet‐derived growth factor; TGF‐β, transforming growth factor‐β; VCAM‐1, vascular cell adhesion molecule 1; VEGF, vascular endothelial growth factor.

The SSc vasculopathy is primarily manifested in capillaries, followed by progressive fibrosis and leading to local tissue ischemia with recurrent episodes of reperfusion.^12^ The myofibroblast differentiation that occurs in response to these initiating microvascular processes is responsible for the excessive accumulation of extracellular matrix (collagen, fibronectin, glycosaminoglycan, and others) in the SSc‐involved tissues.^15^ The EC injury is associated with functional microvascular alterations, like the Raynaud disease, with mechanisms that occur at an early stage of SSc pathogenesis and may be identified morphologically and from the beginning by nailfold videocapillaroscopy (NVC).^2^, ^16^ NVC, a reliable, safe, and highly sensitive method for both qualitative and quantitative evaluation of microvascular status (scleroderma NVC progressive patterns: “very early,” “early,” “active,” or “late”) linked to the severity of the progressive pathophysiologic process and related to organ involvement and/or damage, enables the timely diagnosis (differential) and follow‐up of SSc (Figure 1).^17^, ^18^, ^19^

Microvessels in the skin and visceral organs, being part of the local immune or inflammatory response, undergo changes that lead to progressive tissue remodeling with capillary density decrease and organ function loss.^2^, ^20^ A number of cellular mechanisms drive the transition of ECs to myofibroblasts and fibrosis. Interestingly, significantly increased choroidal thickness and lower choroidal perfusion has been recently and mainly observed by optical coherence tomography angiography (OCT‐A) in patients with diffuse cutaneous SSc (dcSSc) that seems to be related to local subendothelial extracellular matrix deposition.^21^ In addition, the combined analysis of choroidal perfusion by OCT‐A with fingertip perfusion by laser speckle contrast analysis (LASCA) seems to offer preliminary insights that may further complement traditional diagnostic microvascular methods for SSc.

### The cerebrovascular involvement in SSc

**Figure 2:**
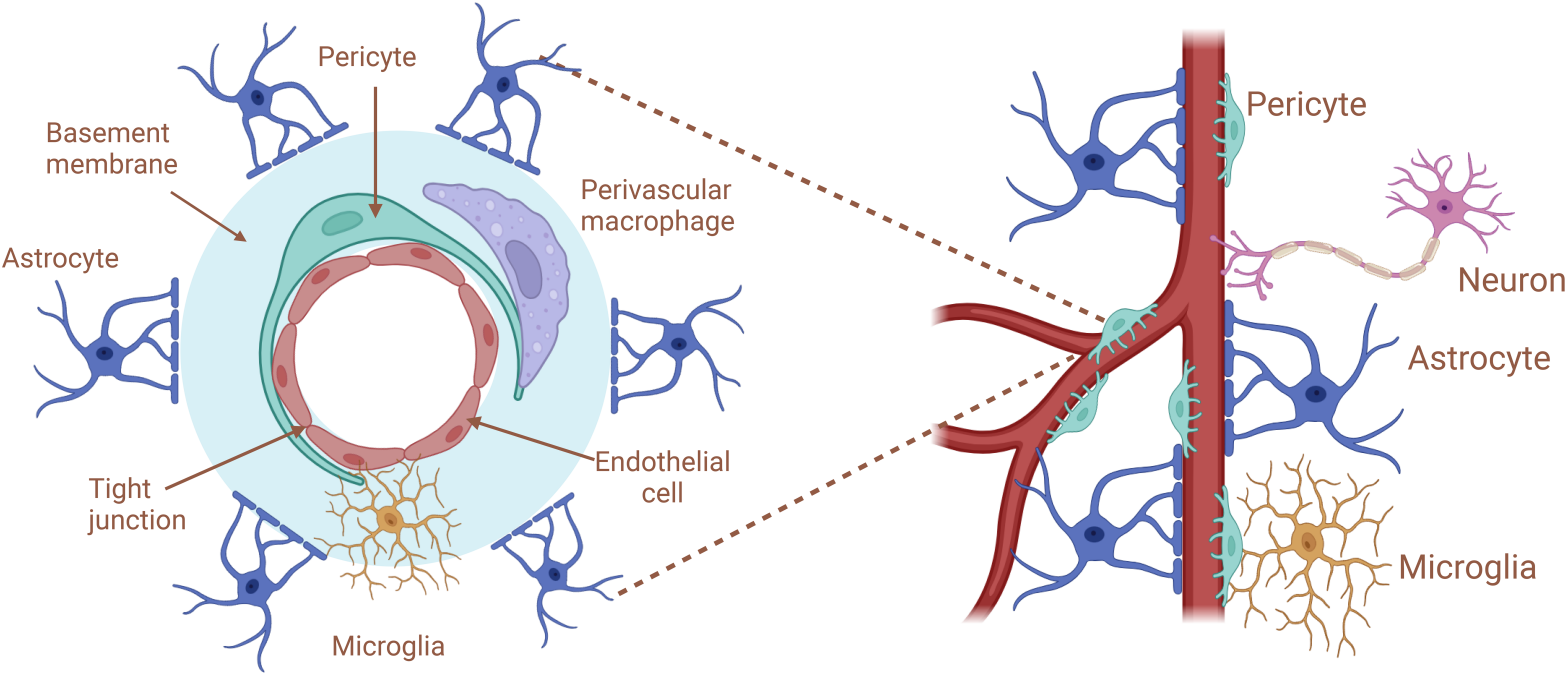
Neurovascular unit: cellular and structural components. Original figure created with BioRender.com.

Up to today, the exact extent and mechanisms underlying CNS involvement in SSc remain incompletely understood, even if vascular abnormalities, inflammation, and autoimmunity are thought to play pivotal roles in the pathogenesis. Blood vessels and nerves share some structural and anatomic similarities and also communicate with each other in a neurovascular crosstalk in which the vessels produce signals that attract axons to develop alongside the pioneer vessels and nerves release local signals that address blood vessel's growth (Figure 2).^22^

In addition, the cerebrovascular system is a highly dynamic multicellular structure that can integrate and respond to both neural and systemic signals. Therefore, the neurovascular unit is a tightly connected and highly coordinated structure that includes ECs and pericytes at the level of capillaries as well as vascular smooth muscle cells at the arterial level and astrocytes, microglia, and neurons.^23^

Therefore, dysfunction and miscommunication among all these different cell types that maintain CNS homeostasis play a crucial role in different neurologic diseases and selectively prevent the entry of several substances from the peripheral blood into the CNS.^24^ Furthermore, brain ECs are more damageable during ischemic conditions than peripheral ECs and generally by inadequate vascular supply, as well as damage of the BBB or neurovascular uncoupling, which supports neuroimmunodegenerative disorders including ischemic white matter lesions.^25^

**Figure 3:**
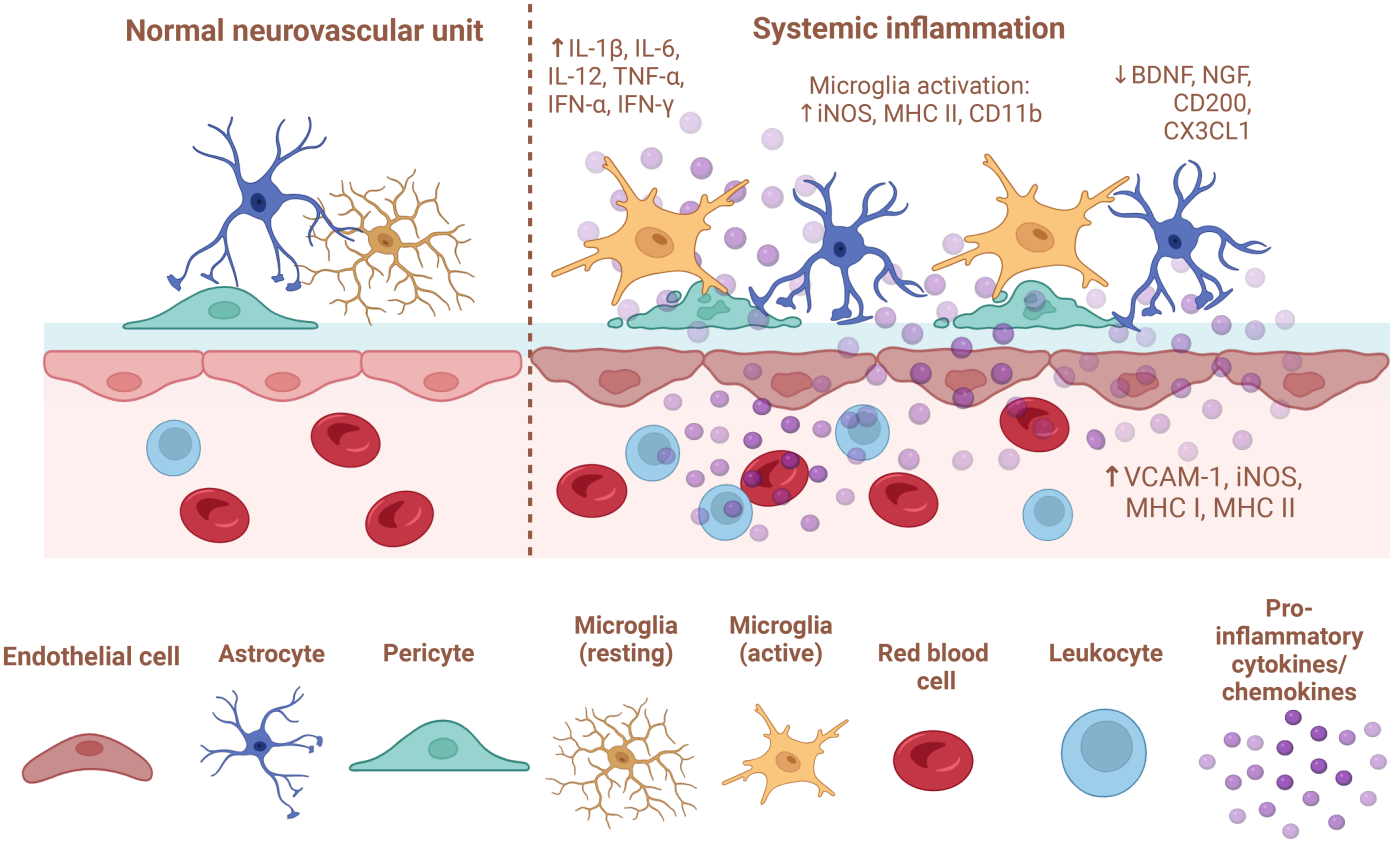
Neurovascular unit at rest and during a systemic inflammatory process. Original figure created with BioRender.com. ↑, increased; ↓, decreased. BDNF, brain‐derived neurotrophic factor; CX3CL1, C‐X3‐C motif chemokine ligand 1; IFN‐α, interferon‐α; IFN‐γ, interferon‐γ; IL‐1β, interleukin‐1β; IL‐6, interleukin‐6; IL‐12, interleukin‐12; iNOS, inducible nitric oxide synthase; MHC I, major histocompatibility complex class I; MHC II, major histocompatibility complex class II; NGF, nerve growth factor; TNF‐α, tumor necrosis factor‐α; VCAM‐1, vascular cell adhesion molecule 1.

Part of the protective function exerted by the BBB includes a significant crosstalk with perivascular macrophages (Ms) and the microglia, which is constituted by the resident Ms in the brain. Therefore, alterations in systemic metabolic and immune‐inflammatory conditions that can cause ECs dysfunction, with reduced integrity of the BBB, are potentially letting peripheral blood mediators and/or substances leak also into the CNS.^25^ Substantially, the process may induce activation of perivascular Ms but also brain microglia and astrocytes and can start further local immune responses within the brain, finally suggesting how neuroinflammation can be triggered by signals and/or mediators (ie, proinflammatory cytokines such as interleukin‐6 [IL‐6], tumor necrosis factor‐α [TNF‐α], and IL‐1β) coming from the periphery, like in the course of systemic ARDs and excluding a primary injury or disease originating within the CNS (Figure 3).^26^, ^27^ In addition and of note, in SSc, the use of a simultaneous in vivo imaging system has shown that systemic inflammation can induce a chemokine receptor (CCR5)–dependent migration of brain resident microglia to the cerebral vasculature.^28^

At the beginning of the inflammatory process, the vessel‐associated microglia keep the BBB integrity and make physical contact with ECs, whereas during sustained inflammation, microglia phagocytose astrocytic end‐feet, impairing the function of the BBB. However, prevalence, severity, and role of vascular involvement of CNS in patients with SSc remains a matter of discussion and is still commonly perceived as rare or as a secondary outcome linked to comorbidities such as hypertension, uremia, pulmonary issues, or even glucocorticoid treatment.^29^ Additionally, occasional transient ischemic attacks, as well as more severe ischemic strokes, and hemorrhages have been recognized as direct consequences of SSc (Table 1).^29^

**Table 1:** CNS involvement in systemic sclerosis*

Study	Population (number of patients)	Nervous system involvement
Gamal et al^29^	72	multiple hyperintense lesions on flair MRI with no diffusion abnormalities (33%)
Mohammed et al^31^	30	MRI white matter focal hyperintense lesions (deep and subcortical regions), also present in brain stem
Sardanelli et al^48^	14	MRI white matter focal hyperintensities: major (>2 mm in diameter) and minor (<2 mm)
Nobili et al^9^	23	MRI white matter focal hyperintensities with CNS hypoperfusion (30%)
Ying et al^34^	4,545	ischemic events (20%–30% higher incidence than in general population)
Rosso et al^5^	58	IS (20.7%), vasculopathy (15.5%), ICH (6.9%), SAH (5.2%), intracranial aneurisms (6.9%)
Jabre et al^52^	11	multiple intracranial aneurysms (55%), SAH (45%)
Kister et al^64^	54	localization‐related epilepsy (73%), MRI focal hyperintensities mostly in subcortical white matter but also in corpus callosum (90%), calcifications (37%), neuropsychological symptoms (15%), headache (35%), vascular changes suggestive of vasculitis (40%)
Amaral et al^32^	9,506	headache (24%), seizures (14%), cognitive impairment (8%), depression (73%), anxiety (24%)
Casale et al^62^	35	subclinical trigeminal damage (17%) associated with central lesion (at medullary or brain level)

* CNS, central nervous system; ICH, intracerebral hemorrhage; IS, ischemic stroke; MRI, magnetic resonance imaging; SAH, subarachnoid hemorrhage.

Diagnosing CNS involvement in SSc offers challenges due to the variety of symptoms and even for the overlap with other neurologic conditions. Indeed, the endothelium damage promoted by inflammatory mediators (IL‐6, TNF‐α, IL‐1β, and CCR5) and the progressive fibrotic process may contribute to cerebrovascular events observed in patients with SSc.

In conclusion, several symptoms of CNS involvement seem supported by microvascular alterations in patients with SSc. Indeed, the recent combined analysis between eye choroidal perfusion by OCT‐A and LASCA has shown a possible way to couple systemic and brain microcirculation.^21^ This concept of considering the eye microcirculation analysis a “window to the brain” is even supported by another interesting study that evaluated the blood flow within the optic nerve head with an ocular laser doppler flowmeter.^30^

### Cerebrovascular disease and ischemic stroke in patients with SSc

The CNS involvement in patients with SSc has traditionally been considered uncommon and even limited because it is attributed to distinctive anatomic features of brain parenchyma (limited profibrotic cells) that render the CNS a difficult target for the development of fibrosing and scarring mechanisms typically seen in all the other organs of such patients.^7^ The proliferation of specific fibroblast subpopulations and invasion into tissue and fibrosis is unlikely to produce CNS abnormalities because of the paucity of connective tissue in the brain.^31^ Furthermore, case reports detailing cerebrovascular disease (CBVD) in patients with SSc have been long regarded as primarily incidental, iatrogenic, or secondary to other manifestations or treatments of SSc.^32^

Conversely, recent meta‐analyses indicate that patients with SSc may indeed have an increased risk of CBVD, irrespective of traditional cardiovascular risk factors, but robust longitudinal studies should be performed to clearly investigate this association and to assess the entity of such risk.^33^, ^34^ In fact, together with microvasculature involvement, SSc has been increasingly linked to macrovascular disease in the form of CBVD and end‐organ manifestations, like pulmonary hypertension and scleroderma renal crisis, that have been found responsible for 20% to 30% of deaths.^35^ Of note, among the causes of brain damage in SSc, ischemic stroke is the most severe cerebrovascular condition associated with the disease and present in around 20.7% of cases (Table 1).^5^

A recent meta‐analysis showed that patients with SSc have at least 1.5 times higher risk of stroke compared with the general population.^36^ In addition, another meta‐analysis found that the risk of stroke (adjusted hazard ratio [HR] 2.35, 95% confidence interval [CI] 1.59–3.48) as well as myocardial infarction (adjusted HR 3.49, 95% CI 2.52–4.83) is the highest during the first year after SSc diagnosis and remains significantly high even after 5 years for both kinds of outcomes.^37^ Furthermore, despite the increased risk of developing a stroke in SSc, no difference was found in stroke outcomes compared with the general population.^38^

Usually, patients with SSc are affected by CBVD in their late 40s, suggesting SSc as a possible risk factor for an earlier onset of brain involvement.^5^ For example, the investigation by Uddin et al concluded that patients with SSc hospitalized due to scleroderma renal crisis showed a significantly increased risk of concurrent acute ischemic stroke versus nonscleroderma renal crisis in patients with SSc.^39^ This observation suggests potential shared pathophysiologic mechanisms between renal and cerebral vasculopathy in patients with SSc.

**Figure 4:**
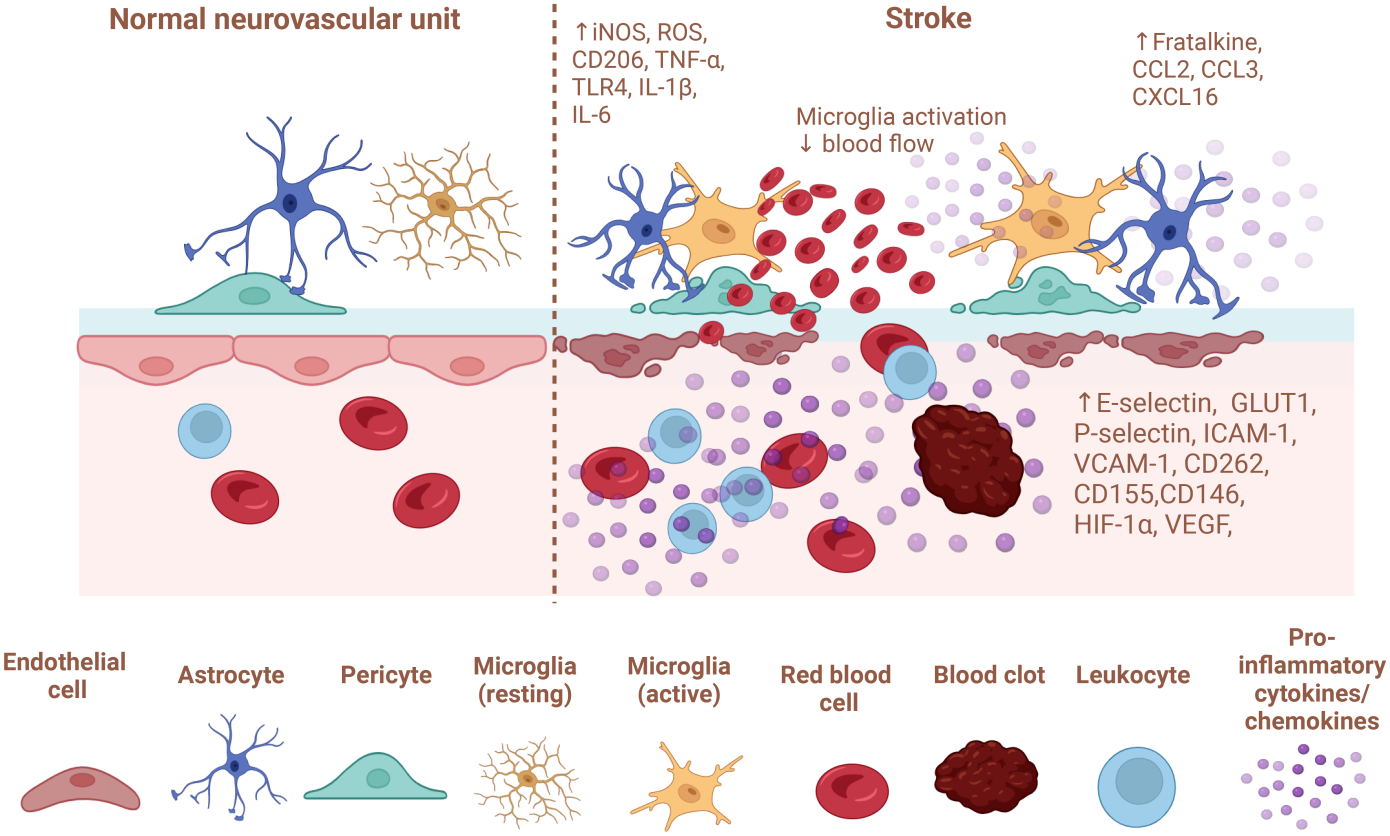
Neurovascular unit at rest and during ischemic stroke. Original figure created with BioRender.com. ↑, increased. CX3CL1, fractalkine; CXCL16, C‐X‐C motif chemokine ligand 16; E‐selectin, endothelial‐selectin; GLUT1, glucose transporter 1; HIF‐1α, hypoxia‐inducible factor 1α; ICAM‐1, intercellular adhesion molecule 1; IL‐1β, interleukin‐1β; IL‐6, interleukin‐6; iNOS, inducible nitric oxide synthase; ROS, reactive oxygen species; TLR4, Toll‐like receptor 4; TNF‐α, tumor necrosis factor‐α; P‐selectin, platelet‐selectin; VCAM‐1, vascular cell adhesion molecule 1; VEGF, vascular endothelial growth factor.

Several possible mechanisms for macrovascular disease in SSc and related CBVD have been considered, including the central role of ECs, involved in both proliferative as well as obliterative and destructive vascular features.^40^ Cerebral vasospasm as evidenced by angiography is another posited mechanism of CBVD in SSc.^41^ In fact, the reversibility of the arterial lesions and the absence of specific histologic findings or association with severe peripheral vascular damage support the important role for vasospasm. In addition, the reported efficacy of immunosuppressive treatments further suggests an association with inflammatory or immune‐mediated mechanisms. Indeed, specific depletion of resident microglia in the early stage of stroke reduces cerebral ischemic damage, even if microglia contribute significantly to the proinflammatory reaction early after the onset of stroke.^42^ Furthermore, the inflammation after stroke contributes to secondary cell injury, mediated at least in part by the inflammasome formation in local cell populations such as microglia, Ms, and ECs themselves.^43^ Therefore, brain inflammasome activation induces a cascade of several inflammatory effects that perpetuate proinflammatory cerebral cell responses (Figure 4).

Several studies have also identified a significant increased occurrence of subclinical cerebrovascular atherosclerosis in general in ARDs versus controls, by measuring carotid intimal medial thickness (CIMT) on ultrasonography and suggesting an elevated risk of stroke.^44^ A total of 68 controlled comparisons from 60 different studies analyzed 25 studies on RA (37%), 24 on SLE (35%), 6 on SSc (9%), and 13 (9%) on other ARDs. Accelerated atherosclerosis was found to be a significantly frequent complication of ARD in 59% of studies, with early alterations visible even in pediatric patients. Interestingly, meta‐analysis reporting the effect size (Cohen's D) of the difference in CIMT between patients with ARDs and control subjects showed SSc comparisons to be the largest (0.58; 95% CI 0.15–1.00).^44^ Recently, a vasculopathy characterized by vessel wall abnormalities on imaging without pathologic tissue confirmation, with or without the presence of ischemic stroke, was found to occur in around 20.7% of patients with SSc and most frequently involves the anterior circulation and frontal lobes (Table 1).^5^ The same findings were reported independently from the different subtypes of SSc, namely limited SSc or dcSSc. Furthermore, structural changes to the blood vessels, particularly fibrous thickening of the intima of small arteries and arterioles, are considered to play a key role in causing vascular dementia.^45^ Vascular alterations are often the cause of neuropsychiatric manifestations in SSc and tend to worsen with disease progression. The clinical manifestations of CNS involvement may range from subtle cognitive impairment, memory loss up to dementia as shown by altered brain function, and decreased spontaneous activity in patients with SSc.^46^

On the other hand, the involvement of the intracranial brain vasculature is thought to arise from vasculopathy contiguous with the overlying subcutaneous localized scleroderma of the head area in patients with SSc.^5^ The presence of both skin scalp atrophy and lobar encephalomalacia is suggestive for a concomitant vasculopathic process in the underlying vasculature of the brain.^5^

### Imaging detection of cerebrovascular involvement in SSc

Pioneering studies in 1997 quantified regional cerebral blood flow with 133Xenon clearance technique in patients with SSc without severe systemic clinical complications and with different severities of peripheral microvascular damage, as assessed by NVC.^9^ Interestingly, in these studies, more significant CNS hypoperfusion was found in the presence of most severe NVC patterns (“active” and “late”) that are associated with advanced SSc. Indeed, the study also detailed the associated extent of the peripheral concomitant microvascular damage, as assessed for the first time, with defined and quantified NVC SSc patterns (“early,” “active,” and “late”).^9^ In particular, cerebral hypoperfusion was found in 52% of patients with SSc, namely in 33% of patients with the “early” NVC pattern, in 56% of patients with the “active” pattern, and 67% of patients showing the “late” NVC pattern. Furthermore, at magnetic resonance imaging (MRI) analysis, white matter focal and/or diffuse signal abnormalities in both hemispheres were found at the highest rate (44%) in patients with SSc with the “late” NVC pattern.^9^

The evidence of cerebral hypoperfusion in patients with SSc was revisited by the same team^10^ in 2000. Interestingly, the investigation reported local or diffuse cerebral hypoperfusion in more than half of the neurologically asymptomatic patients with SSc evaluated, paralleling the incidence of altered brain MRI. Of note, hypoperfusion was not linked to aging and possibly was suggested to reflect the brain location of the microangiopathic process characterizing the systemic disease. In addition, brain functional microvascular deficits, characterized by focal and diffuse cerebral hypoperfusion, were again found in almost half of patients with SSc, mainly neurologically asymptomatic, by using this time the single photon emission computed tomography (SPECT) with perfusion tracers that can disclose regional hypoperfusion (Table 1).^47^

Again, in the early 2000s, some MRI studies reported that vascular CNS involvement in SSc is not uncommon, and these features might be asymptomatic and independent of disease duration.^48^ Indeed, CNS involvement in the form of significant white matter hyperintense foci in the deep subcortical regions versus healthy controls was again confirmed in patients with SSc on MRI evaluation, indicating a form of brain vasculopathy, and moreover, the observation showed a significant correlation to the severity of peripheral vascular disease.^49^ Very recently, the changes of cerebral structure and perfusion evaluated by MRI were shown to vary among different disease subtypes of SSc, with higher severity in patients with dcSSc.^50^

In addition, more severe skin fibrosis seems to indicate a higher risk of brain involvement in patients with SSc and is usually associated with advanced miscrovascular damage as detected by NVC (“late” pattern).^17^ Interestingly, calcifications in small arteries and arterioles, primarily in the basal ganglia but also in the frontal lobe and in the cerebellar area, were found in postmortem brain examinations of patients with SSc and confirmed in other patients by a prospective computed tomography study, confirming that SSc may induce vascular remodeling in the brain.^51^ Intracerebral calcifications were found significantly associated with the duration of Raynaud disease but not with age. Of course, together with neuroimaging, cerebrospinal fluid analysis and neuropsychological assessments should be instrumental in the differential diagnostic process.

### Further and rare conditions of cerebrovascular involvement in SSc

As mentioned, intracerebral hemorrhage (ICH) has been reported in patients with SSc presenting minimal ICH risk factors, but whether SSc‐mediated vasculopathy also independently increases the risk of ICH, similarly to ischemic stroke, remains unknown (Table 1).^29^ The EC injury in SSc may lead to the formation of intracranial aneurysms, also recognized to be associated with other vascular diseases. A systematic review found that two‐thirds of patients with SSc with aneurysms showed multiple aneurysms (≥2 cm), and 12% were giant (≥2.5 cm); in addition, nearly half were associated with subarachnoid hemorrhage (SAH) (Table 1).^52^ Recently, four patients have been diagnosed with SSc and angiographically proven Moya‐Moya syndrome, a rare cerebrovascular disorder that affects the internal carotid arteries, leading us to hypothesize several mechanisms, including vasculitis and ECs hyperplasia.^50^ It is thought that the presence of peripheral vascular disease in patients with SSc may potentially support future Moya‐Moya syndrome, and early MR angiography screening may be important for identifying and addressing progressive vaso‐occlusive disease for optimal management.^53^

A Raynaud‐like phenomenon, with Raynaud being the most frequent early clinical manifestation of SSc, can also occur in the brain, causing reversible cerebral vasoconstriction syndrome (RCVS), a neurovascular disorder characterized by a reversible spasm of cerebral arteries.^54^ Few cases of association between RCVS and SSc have been reported.^55^, ^56^ However, the incidence of RCVS in the context of autoimmune disorders is rarely reported, and the exact pathophysiology of RCVS is not fully elucidated, but several risk factors are known including the administration of high‐dose glucocorticoids.^57^ Furthermore, RCVS may contribute to ICH and SAH, thus increasing the morbidity of SSc.^58^

### PNS involvement in patients with SSc

Peripheral neuropathy that is mostly detected in the first decade of the SSc course encompasses a broad array of clinical conditions impacting sensory, motor, and autonomic nerve fibers and has a number of different etiologies, including nerve compression by soft tissue swelling, tissue fibrosis or calcinosis cutis, traumatic injury, medication adverse effects, metabolic sequelae, and ischemia.^32^ Peripheral neuropathies in patients with SSc are also defined by the extent of nerve fiber involvement, ranging from single nerve involvement (mononeuropathies) to multiple nerves affected individually (mononeuritis multiplex syndrome). This wide spectrum of possible etiologies can make the diagnosis of peripheral neuropathy challenging.^59^

Various risk factors and causes have been linked to the development of noncompression peripheral neuropathy in SSc. Indeed, complications stemming from organ damage in ARDs, such as gastrointestinal tract involvement with malabsorption and consequent myelopathy, as well as vitamin deficiencies (vitamin E, cyanocobalamin, or calciferol), direct traumatic injury, medication adverse effects, ischemia, or renal crisis, leading to hypertensive and/or uremic neuropathy, can result in peripheral neuropathy.^60^

A symmetric sensory polyneuropathy is the most frequently associated noncompression neuropathy in patients with SSc, whereas the peripheral polyneuropathies (mixed sensory and motor) and mononeuritis multiplex are more common in overt SSc. A large systematic review including 9,506 patients with SSc with neurologic manifestations revealed that the peripheral sensory and/or motor polyneuropathy was present in 14.25% of the patients but also identified carpal tunnel syndrome in 6.56%, ulnar neuropathy in 3.39%, and multiple mononeuropathies in 1.81% of patients with SSc (Table 1).^32^

On the other hand, the most common cranial neuropathies in SSc seem to include trigeminal neuropathy with dysfunction of the optic, oculomotor, trochlear, abducens, facial, glossopharyngeal, and auditory nerves and can usually precede overt SSc. Roughly 4% of cranial neuropathies are observed in patients with SSc, making such condition the second most prevalent cause linked to autoimmune mechanisms.^61^ The hypothesized underlying pathophysiologic SSc mechanisms may include microvascular altered function and/or local immune response and/or fibrosis that occur in the trigeminal ganglion or root at vasa nervorum level.^62^

The pathophysiology of scleroderma‐associated neuropathy remains to be further investigated but seems to involve the dysregulation of the neuroendothelial control of vascular tone as the leading hypothesis (Raynaud and microvascular abnormalities).^63^ Furthermore, recent investigations identified a group of neurovascular guidance molecules with neurogenic and angiogenic properties (semaphorin/plexin/neuropilin and slit/robo families) that seem to correlate with altered microvascular architecture (as detected by NVC) and ischemic events, suggesting an active role in SSc pathogenesis and disease progression.^63^

### Limitations and future prospective for CNS involvement in SSc

At present, the major limitations for strong cerebrovascular status reports in SSc are due to absence of longitudinal studies with neuroimaging at baseline and during the follow‐up. To date, the most relevant outcomes derive from cross‐sectional studies performing analysis of cerebrovascular status done by MRI or positron emission tomography‐computed tomography. Moreover, although neuroimaging may play a critical role in early detection and evaluation of the extent of CNS involvement, it is not commonly performed in SSc clinical management. Indeed, future endeavors involving new neuroimaging technologies (functional MRI, MR spectroscopy, or SPECT) combined with peripheral microvascular evaluation (ie OCT‐A, LASCA, and NVC) might contribute to more comprehensive detection of the disease status and, eventually, an optimized early treatment for SSc. As a matter of fact, the early use of immunosuppressive drugs in SSc can counteract the systemic immune response and inflammation and has proven to reduce the incidence of stroke.

## Conclusions

The altered function of CNS in patients with SSc related to cerebrovascular involvement is today well documented, often without evident clinical symptoms or signs. However, it may contribute to the complexity of the disease during its progression, impacting both patient quality of life and disease outcome. The use of advanced neuroimaging methods to detect cerebrovascular status is mandatory, even in patients with SSc with minimal signs of CNS involvement or in asymptomatic patients with dcSSc, to identify early structural, vascular, and functional changes in the brain.

Understanding CNS involvement in SSc is crucial for improving diagnostic accuracy, early diagnosis, and possibly the follow‐up of cerebrovascular damage, as well as the development of targeted therapeutic interventions. Currently, there are no specific targeted therapies for CNS involvement in SSc, and the management is primarily based on addressing the underlying autoimmune and/or inflammatory activity and vascular damage. Therefore, in patients with SSc, ongoing immunosuppressive and anti‐inflammatory treatments, including specific cerebrovascular and/or neurologic supportive therapies, still represent the best available therapeutic strategies also for the CNS manifestations. In conclusion, in light of the current state of art, specialists should always consider potential CNS involvement in SSc as part of the disease's clinical complication spectrum to optimize comprehensive patient care.

## AUTHOR CONTRIBUTIONS

All authors contributed to at least one of the following manuscript preparation roles: conceptualization AND/OR methodology, software, investigation, formal analysis, data curation, visualization, and validation AND drafting or reviewing/editing the final draft. As corresponding author, Dr Cutolo confirms that all authors have provided the final approval of the version to be published and takes responsibility for the affirmations regarding article submission (eg, not under consideration by another journal), the integrity of the data presented, and the statements regarding compliance with institutional review board/Declaration of Helsinki requirements.

REFERENCES1

Volkmann
ER
, 
Andréasson
K
, 
Smith
V
. Systemic sclerosis. Lancet
2023;401(10373):304–318.36442487
10.1016/S0140-6736(22)01692-0PMC98923432

Cutolo
M
, 
Smith
V
. Detection of microvascular changes in systemic sclerosis and other rheumatic diseases. Nat Rev Rheumatol
2021;17(11):665–677.34561652
10.1038/s41584-021-00685-03

Juncker
AS
, 
Appenzeller
S
, 
de Souza
JM
. Central nervous system involvement in systemic autoimmune rheumatic diseases‐diagnosis and treatment. Pharmaceuticals (Basel)
2024;17(8):1044.39204149
10.3390/ph17081044PMC113574374

Kandemirli
SG
, 
Bathla
G
. Neuroimaging findings in rheumatologic disorders. J Neurol Sci
2021;427:117531.34130065
10.1016/j.jns.2021.1175315

Rosso
M
, 
Ramaswamy
S
, 
Aharonoff
D
, et al. Spectrum of cerebrovascular disease in scleroderma: a case series and systematic review. Cerebrovasc Dis
2023;53(4):467–478.37839405
10.1159/0005332306

Pope
JE
, 
Denton
CP
, 
Johnson
SR
, et al. State‐of‐the‐art evidence in the treatment of systemic sclerosis. Nat Rev Rheumatol
2023;19(4):212–226.36849541
10.1038/s41584-023-00909-5PMC99701387

Averbuch‐Heller
L
, 
Steiner
I
, 
Abramsky
O
. Neurologic manifestations of progressive systemic sclerosis. Arch Neurol
1992;49(12):1292–1295.1333182
10.1001/archneur.1992.005303600940248

Wuriliga
DX
, 
Xu
D
, 
He
Y
, et al. Mild cognitive impairment in patients with systemic sclerosis and features analysis. Rheumatology (Oxford)
2022;61(6):2457–2463.39806733
10.1093/rheumatology/keab7879

Nobili
F
, 
Cutolo
M
, 
Sulli
A
, et al. Impaired quantitative cerebral blood flow in scleroderma patients. J Neurol Sci
1997;152(1):63–71.9395127
10.1016/s0022-510x(97)00144-510

Cutolo
M
, 
Nobili
F
, 
Sulli
A
, et al. Evidence of cerebral hypoperfusion in scleroderma patients. Rheumatology (Oxford)
2000;39(12):1366–1373.11136880
10.1093/rheumatology/39.12.136611

Smith
V
, 
Vanhaecke
A
, 
Herrick
AL
, et al; EULAR Study Group on Microcirculation in Rheumatic Diseases. Fast track algorithm: how to differentiate a “scleroderma pattern” from a “non‐scleroderma pattern”. Autoimmun Rev
2019;18(11):102394.31520797
10.1016/j.autrev.2019.10239412

Cutolo
M
, 
Soldano
S
, 
Smith
V
. Pathophysiology of systemic sclerosis: current understanding and new insights. Expert Rev Clin Immunol
2019;15(7):753–764.31046487
10.1080/1744666X.2019.161491513

Hysa
E
, 
Pizzorni
C
, 
Sammorì
S
, et al. Microvascular damage in autoimmune connective tissue diseases: a capillaroscopic analysis from 20 years of experience in a EULAR training and research referral centre for imaging. RMD Open
2023;9(3):e003071.37451812
10.1136/rmdopen-2023-003071PMC1035126914

Marongiu
F
, 
Ruberto
MF
, 
Marongiu
S
, et al. A journey to vasculopathy in systemic sclerosis: focus on haemostasis and thrombosis. Clin Exp Med
2023;23(8):4057–4064.37914967
10.1007/s10238-023-01222-x15

Ho
YY
, 
Lagares
D
, 
Tager
AM
, et al. Fibrosis‐‐a lethal component of systemic sclerosis. Nat Rev Rheumatol
2014;10(7):390–402.24752182
10.1038/nrrheum.2014.5316

Pacini
G
, 
Pogna
A
, 
Pendolino
M
, et al. Understanding the value of non‐specific abnormal capillary dilations in presence of Raynaud's phenomenon: a detailed capillaroscopic analysis. RMD Open
2022;8(2):e002449.36197673
10.1136/rmdopen-2022-002449PMC946209317

Sulli
A
, 
Paolino
S
, 
Pizzorni
C
, et al. Progression of nailfold capillaroscopic patterns and correlation with organ involvement in systemic sclerosis: a 12 year study. Rheumatology (Oxford)
2020;59(5):1051–1058.31750929
10.1093/rheumatology/kez37418

Smith
V
, 
Herrick
AL
, 
Ingegnoli
F
, et al; EULAR Study Group on Microcirculation in Rheumatic Diseases and the Scleroderma Clinical Trials Consortium Group on Capillaroscopy. Standardisation of nailfold capillaroscopy for the assessment of patients with Raynaud's phenomenon and systemic sclerosis. Autoimmun Rev
2020;19(3):102458.31927087
10.1016/j.autrev.2020.10245819

Smith
V
, 
Ickinger
C
, 
Hysa
E
, et al. Nailfold capillaroscopy. Best Pract Res Clin Rheumatol
2023;37(1):101849.37419757
10.1016/j.berh.2023.10184920

Ruaro
B
, 
Sulli
A
, 
Pizzorni
C
, et al. Correlations between blood perfusion and dermal thickness in different skin areas of systemic sclerosis patients. Microvasc Res
2018;115:28–33.28834709
10.1016/j.mvr.2017.08.00421

Cutolo
CA
, 
Cere
A
, 
Toma
P
, et al. Peripheral and ocular microvascular alterations in systemic sclerosis: observations from capillaroscopic assessments, perfusion peripheral analysis, and optical coherence tomography angiography. Rheumatol Int
2024;44(1):107–118.37978075
10.1007/s00296-023-05495-zPMC1076677822

Romano
E
, 
Rosa
I
, 
Fioretto
BS
, et al. Circulating neurovascular guidance molecules and their relationship with peripheral microvascular impairment in systemic sclerosis. Life (Basel)
2022;12(7):1056.35888144
10.3390/life12071056PMC931634323

De Luca
C
, 
Colangelo
AM
, 
Virtuoso
A
, et al. Neurons, glia, extracellular matrix and neurovascular unit: a systems biology approach to the complexity of synaptic plasticity in health and disease. Int J Mol Sci
2020;21(4):1539.32102370
10.3390/ijms21041539PMC707323224

Winkler
EA
, 
Bell
RD
, 
Zlokovic
BV
. Central nervous system pericytes in health and disease. Nat Neurosci
2011;14(11):1398–1405.22030551
10.1038/nn.2946PMC402062825

Mayer
MG
, 
Fischer
T
. Microglia at the blood brain barrier in health and disease. Front Cell Neurosci
2024;18:1360195.38550920
10.3389/fncel.2024.1360195PMC1097685526

Sanmarco
LM
, 
Polonio
CM
, 
Wheeler
MA
, et al. Functional immune cell‐astrocyte interactions. J Exp Med
2021;218(9):e20202715.34292315
10.1084/jem.20202715PMC830244727

Knopp
RC
, 
Banks
WA
, 
Erickson
MA
. Physical associations of microglia and the vascular blood‐brain barrier and their importance in development, health, and disease. Curr Opin Neurobiol
2022;77:102648.36347075
10.1016/j.conb.2022.10264828

Haruwaka
K
, 
Ikegami
A
, 
Tachibana
Y
, et al. Dual microglia effects on blood brain barrier permeability induced by systemic inflammation. Nat Commun
2019;10(1):5816.31862977
10.1038/s41467-019-13812-zPMC692521929

Gamal
RM
, 
Abozaid
HSM
, 
Zidan
M
, et al. Study of MRI brain findings and carotid US features in systemic sclerosis patients, relationship with disease parameters. Arthritis Res Ther
2019;21(1):95.30987675
10.1186/s13075-019-1877-zPMC646674230

Nenekidis
I
, 
Geiser
M
, 
Riva
C
, et al. Blood flow measurements within optic nerve head during on‐pump cardiovascular operations. A window to the brain?
Interact Cardiovasc Thorac Surg
2011;12(5):718–722.21297131
10.1510/icvts.2010.26095031

Mohammed
RH
, 
Sabry
YY
, 
Nasef
AA
. Brain MRI screening showing evidences of early central nervous system involvement in patients with systemic sclerosis. Rheumatol Int
2011;31(5):667–671.20069332
10.1007/s00296-009-1325-532

Amaral
TN
, 
Peres
FA
, 
Lapa
AT
, et al. Neurologic involvement in scleroderma: a systematic review. Semin Arthritis Rheum
2013;43(3):335–347.23827688
10.1016/j.semarthrit.2013.05.00233

Ungprasert
P
, 
Sanguankeo
A
, 
Upala
S
. Risk of ischemic stroke in patients with systemic sclerosis: a systematic review and meta‐analysis. Mod Rheumatol
2016;26(1):128–131.26025436
10.3109/14397595.2015.105693134

Ying
D
, 
Gianfrancesco
MA
, 
Trupin
L
, et al. Increased risk of ischemic stroke in systemic sclerosis: a national cohort study of US veterans. J Rheumatol
2020;47(1):82–88.30877213
10.3899/jrheum.181311PMC726919935

Hettema
ME
, 
Bootsma
H
, 
Kallenberg
CG
. Macrovascular disease and atherosclerosis in SSc. Rheumatology (Oxford)
2008;47(5):578–583.18321944
10.1093/rheumatology/ken07836

Chen
IW
, 
Wang
WT
, 
Lai
YC
, et al. Association between systemic sclerosis and risk of cerebrovascular and cardiovascular disease: a meta‐analysis. Sci Rep
2024;14(1):6445.38499699
10.1038/s41598-024-57275-9PMC1094890437

Aviña‐Zubieta
JA
, 
Man
A
, 
Yurkovich
M
, et al. Early cardiovascular disease after the diagnosis of systemic sclerosis. Am J Med
2016;129(3):324–331.26603342
10.1016/j.amjmed.2015.10.03738

Edigin
E
, 
Eseaton
P
, 
Kaul
S
, et al. Systemic sclerosis is not associated with worse outcomes of patients admitted for ischemic stroke: analysis of the National Inpatient Sample. Cureus
2020;12(7):e9155.32789091
10.7759/cureus.9155PMC741732139

Uddin
M
, 
Mir
T
, 
Surapaneni
S
, et al. Scleroderma hypertensive renal crisis among systemic sclerosis patients: a National Emergency Department Database study. Am J Emerg Med
2022;53:228–235.35078051
10.1016/j.ajem.2022.01.02040

Asano
Y
, 
Sato
S
. Vasculopathy in scleroderma. Semin Immunopathol
2015;37(5):489–500.26152638
10.1007/s00281-015-0505-541

Faucher
B
, 
Granel
B
, 
Nicoli
F
. Acute cerebral vasculopathy in systemic sclerosis. Rheumatol Int
2013;33(12):3073–3077.23263549
10.1007/s00296-012-2614-y42

Chen
AQ
, 
Fang
Z
, 
Chen
XL
, et al. Microglia‐derived TNF‐α mediates endothelial necroptosis aggravating blood brain‐barrier disruption after ischemic stroke. Cell Death Dis
2019;10(7):487.31221990
10.1038/s41419-019-1716-9PMC658681443

Bellut
M
, 
Papp
L
, 
Bieber
M
, et al. NLPR3 inflammasome inhibition alleviates hypoxic endothelial cell death in vitro and protects blood‐brain barrier integrity in murine stroke. Cell Death Dis
2021;13(1):20.34930895
10.1038/s41419-021-04379-zPMC868841444

Tyrrell
PN
, 
Beyene
J
, 
Feldman
BM
, et al. Rheumatic disease and carotid intima‐media thickness: a systematic review and meta‐analysis. Arterioscler Thromb Vasc Biol
2010;30(5):1014–1026.20150560
10.1161/ATVBAHA.109.19842445

O'Brien
JT
, 
Thomas
A
. Vascular dementia. Lancet
2015;386(10004):1698–1706.26595643
10.1016/S0140-6736(15)00463-846

Tong
X
, 
He
H
, 
Xu
S
, et al. Brain functional alternation in patients with systemic sclerosis: a resting‐state functional magnetic resonance imaging study. Arthritis Res Ther
2024;26(1):194.39516849
10.1186/s13075-024-03433-3PMC1154531447

Nobili
F
, 
Cutolo
M
, 
Sulli
A
, et al. Brain functional involvement by perfusion SPECT in systemic sclerosis and Behçet's disease. Ann NY Acad Sci
2002;966(1):409–414.12114298
10.1111/j.1749-6632.2002.tb04241.x48

Sardanelli
F
, 
Iozzelli
A
, 
Cotticelli
B
, et al. White matter hyperintensities on brain magnetic resonance in systemic sclerosis. Ann Rheum Dis
2005;64(5):777–779.15834058
10.1136/ard.2003.018283PMC175550049

Mohamed
RH
, 
Nassef
AA
. Brain magnetic resonance imaging findings in patients with systemic sclerosis. Int J Rheum Dis
2010;13(1):61–67.20374386
10.1111/j.1756-185X.2009.01453.x50

Tong
X
, 
He
H
, 
Xu
S
, et al. Changes of cerebral structure and perfusion in subtypes of systemic sclerosis: a brain magnetic resonance imaging study. Rheumatology (Oxford)
2024;63(12):3263–3270.39102826
10.1093/rheumatology/keae40451

Heron
E
, 
Hernigou
A
, 
Chatellier
G
, et al. Intracerebral calcification in systemic sclerosis. Stroke
1999;30(10):2183–2185.10512926
10.1161/01.str.30.10.218352

Jabre
R
, 
Benomar
A
, 
Bojanowski
MW
. Scleroderma's possible dual role in the pathophysiology of intracranial aneurysms: case report and literature review. World Neurosurg
2020;141:267–271.32461175
10.1016/j.wneu.2020.05.17053

Chen
H
, 
Jiang
X
, 
Shi
Y
, et al. Systemic sclerosis associated with moyamoya syndrome: a case report and literature review. Immunobiology
2020;225(2):151882.31812345
10.1016/j.imbio.2019.11.01754

Gupta
S
, 
Zivadinov
R
, 
Ramasamy
D
, et al. Reversible cerebral vasoconstriction syndrome (RCVS) in antiphospholipid antibody syndrome (APLA): the role of centrally acting vasodilators. Case series and review of literature. Clin Rheumatol
2014;33(12):1829–1833.24277114
10.1007/s10067-013-2434-955

Etemadifar
M
, 
Shafiei
M
, 
Salari
M
, et al. Systemic sclerosis, reversible cerebral vasoconstriction syndrome, and neuromyelitis optica in a patient. Case Reports Immunol
2022;2022:8541329.35864935
10.1155/2022/8541329PMC929634956

Liu
J
, 
Guo
M
, 
Beegle
RD
, et al. A case report of reversible cerebral vasoconstriction syndrome in a patient with systemic scleroderma. Cureus
2022;14(4):e24364.35619870
10.7759/cureus.24364PMC912642757

Singhal
AB
, 
Topcuoglu
MA
. Glucocorticoid‐associated worsening in reversible cerebral vasoconstriction syndrome. Neurology
2017;88(3):228–236.27940651
10.1212/WNL.0000000000003510PMC527279358

Werring
DJ
. Reversible cerebral vasoconstriction syndrome and intracranial hemorrhage: some answers, many questions. Stroke
2010;41(11):2455–2456.20884866
10.1161/STROKEAHA.110.59390559

AlMehmadi
BA
, To FZ
, 
Anderson
MA
, et al. Epidemiology and treatment of peripheral neuropathy in systemic sclerosis. J Rheumatol
2021;48(12):1839–1849.34210833
10.3899/jrheum.20129960

De Souza
JM
, 
Trevisan
TJ
, 
Sepresse
SR
, et al. Peripheral neuropathy in systemic autoimmune rheumatic diseases‐diagnosis and treatment. Pharmaceuticals (Basel)
2023;16(4):587.37111344
10.3390/ph16040587PMC1014198661

Farrell
DA
, 
Medsger
TA
Jr.

Trigeminal neuropathy in progressive systemic sclerosis. Am J Med
1982;73(1):57–62.7091174
10.1016/0002-9343(82)90926-362

Casale
R
, 
Frazzitta
G
, 
Fundarò
C
, et al. Blink reflex discloses CNS dysfunction in neurologically asymptomatic patients with systemic sclerosis. Clin Neurophysiol
2004;115(8):1917–1920.15261870
10.1016/j.clinph.2004.03.00363

Romano
E
, 
Rosa
I
, 
Fioretto
BS
, et al. A new avenue in the pathogenesis of systemic sclerosis: the molecular interface between the endothelial and the nervous systems. Clin Exp Rheumatol
2019;37 Suppl 119(4):133–140.3102593264

Kister
I
, 
Inglese
M
, 
Laxer
RM
, et al. Neurologic manifestations of localized scleroderma: a case report and literature review. Neurology
2008;71(19):1538–1545.18981376
10.1212/01.wnl.0000334474.88923.e3

## Supporting information


**Disclosure form**.
